# RELIGIOUS ATTENDANCE AND SLEEP QUALITY IN US OLDER ADULTS

**DOI:** 10.1093/geroni/igad104.1266

**Published:** 2023-12-21

**Authors:** Katherine Britt, Fanghong Dong, Nancy Hodgson

**Affiliations:** The University of Pennsylvania, Philadelphia, Pennsylvania, United States; University of Pennsylvania, Philadelphia, Pennsylvania, United States; University of Pennsylvania School of Nursing, Philadelphia, Pennsylvania, United States

## Abstract

Sleep is an important proponent of memory, learning, and cognitive processing with changing patterns throughout the life course; poor sleep contributes to cognitive decline and dementia risk. Spiritual and religious activities are associated with better cognitive health, yet the biological mechanism is not well understood. This study aims to investigate whether religious and spiritual activity is associated with sleep quality in U.S. adults. Data were drawn from a U.S. longitudinal survey of a national probability sample, Midlife in the United States (MIDUS), from MIDUS Wave 2 (2004-2006), and MIDUS refresher (2011-2014). We examined cross-sectional associations between self-reported religious service attendance measured with 1 item (high, low religious attendance) and sleep quality measured with the Pittsburgh Sleep Quality Index (PSQI) (n=1,168) across three adult age groups (early, middle, late). We adjusted for sex, education, marital status, BMI, and smoking. Higher religious service attendance was significantly associated with better sleep quality among older adults (aged 60 – 80 years) (B=-1.02, p=0.011), while associations in young (aged 20-40 years) (B=-0.76, p=0.087) and middle-aged adults (aged 40-60 years) (B=-0.54, p=0.061) were marginal. Religious service attendance may contribute to better sleep quality amongst older adults by providing a sense of purpose and reducing feelings of helplessness and a sense of loss of control associated with aging. In addition, religious attendance is associated with decreased depressive symptoms and anxiety, possibly contributing to greater spiritual well-being and promoting better sleep. Longitudinal studies are needed to examine these associations over time.

